# *N*′-[1-(5-Bromo-2-hy­droxy­phen­yl)ethyl­idene]isonicotinohydrazide monohydrate: crystal structure and Hirshfeld surface analysis

**DOI:** 10.1107/S2056989017004790

**Published:** 2017-03-31

**Authors:** See Mun Lee, Nathan R. Halcovitch, Mukesh M. Jotani, Edward R. T. Tiekink

**Affiliations:** a Research Centre for Crystalline Materials, School of Science and Technology, Sunway University, 47500 Bandar Sunway, Selangor Darul Ehsan, Malaysia; b Department of Chemistry, Lancaster University, Lancaster LA1 4YB, United Kingdom; c Department of Physics, Bhavan’s Sheth R. A. College of Science, Ahmedabad, Gujarat 380001, India

**Keywords:** crystal structure, carbohydrazide, hydrogen bonding, halogen bonding, Hirshfeld surface analysis

## Abstract

In the title isonicotinohydrazide hydrate, C_14_H_12_BrN_3_O_2_·H_2_O {systematic name: *N*′-[(1*E*)-1-(5-bromo-2-hy­droxy­phen­yl)ethyl­idene]pyridine-4-carbohydrazide monohydrate}, the central CN_2_O region of the organic mol­ecule is planar and the conformation about the imine-C=N bond is *E*. While an intra­molecular hy­droxy-O—H⋯N(imine) hydrogen bond is evident, the dihedral angle between the central residue and the benzene rings is 48.99 (9)°. Overall, the mol­ecule is twisted, as seen in the dihedral angle of 71.79 (6)° between the outer rings. In the crystal, hydrogen-bonding inter­actions, *i.e*. hydrazide-N—H⋯O(water), water-O—H⋯O(carbon­yl) and water-O—H⋯N(pyrid­yl), lead to supra­molecular ribbons along the *a-*axis direction. Connections between these, leading to a three-dimensional architecture, are mediated by Br⋯Br halogen bonding [3.5366 (3) Å], pyridyl-C—H⋯O(carbon­yl) as well as weak π–π inter­actions [inter-centroid separation between benzene rings = 3.9315 (12) Å]. The Hirshfeld surface analysis reveals the importance of hydrogen atoms in the supra­molecular connectivity as well as the influence of the Br⋯Br halogen bonding.

## Abstract 2

The organic mol­ecule is twisted with a near orthogonal relationship between the outer rings [dihedral angle = 71.79 (6)°]. Supra­molecular ribbons sustained by hydrogen bonding feature in the mol­ecular packing.

## Chemical context   

Schiff bases play an important role in inorganic chemistry as they can easily form stable complexes with metal ions. Schiff base ligands have now been designed that may bind in a variety of modes in their metal complexes, *i.e*. monodentate, bidentate, tridentate and even tetra­dentate. Recent inter­est in the coordination of hydrazide Schiff base ligands arises owing to the presence of electron-donating nitro­gen and oxygen atoms, allowing these to act as a multidentate ligands, and in some cases, function as supra­molecular building blocks in their mol­ecular assemblies (Wei *et al.*, 2015 ▸; Nie & Huang 2006 ▸). In recent years, studies of organotin(IV) compounds has gained inter­est as a result of their potential industrial and biocidal applications (Davies *et al.*, 2008 ▸). Among these compounds, the chemistry and applications of organotin(IV) complexes with Schiff base ligands have been studied extensively due to their structural diversity, thermal stability and biological properties. As part of on-going work with these ONO tridentate ligands (Lee *et al.*, 2012 ▸, 2013 ▸, 2015 ▸), the crystal and mol­ecular structures of the title compound (I), obtained as a side-product during the preparation of an organotin compound, is described along with a detailed evaluation of the inter­molecular association in the crystal through a Hirshfeld surface analysis.
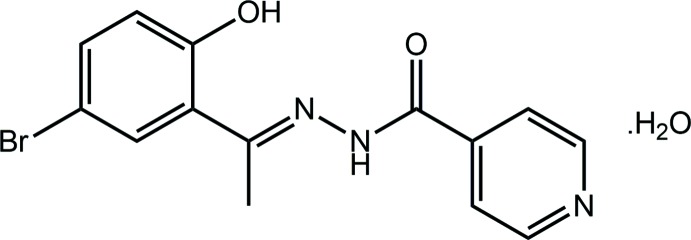


## Structural commentary   

**Figure 1:**
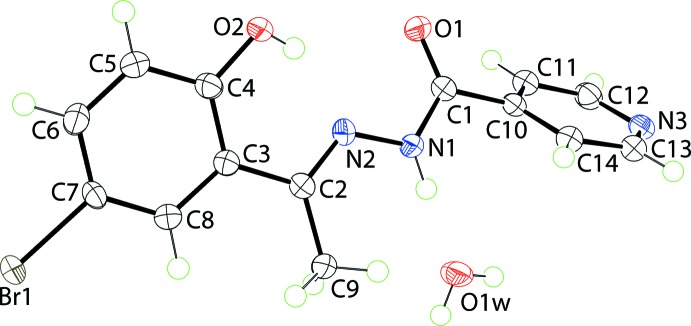
The mol­ecular structures of constituents of (I) showing the atom-labelling scheme and displacement ellipsoids at the 70% probability level.

The mol­ecular structures of the constituents of (I) are shown in Fig. 1 ▸. The organic mol­ecule features a central, essentially planar region flanked on either side by a pyridyl ring and a di-substituted benzene ring. The central residue comprising the N1, N2, O1 and C1 atoms is strictly planar [r.m.s. deviation of the fitted atoms = 0.0001 Å] with the C2 and C10 atoms lying 0.171 (3) and 0.010 (4) Å, respectively, out of the plane; the carbonyl-O and hydrazide-NH groups are *anti*. The sequence of N1—N2—C2—C3 [−177.59 (15)°], N2—N1—C1—C10 [179.59 (15)°] and C1—N1—N2—C2 [171.14 (18)°] torsion angles is consistent with an all-*trans* relationship in the central chain and a small twist about the N1—N2 bond. The conformation about the imine-C2=N2 bond [1.292 (2) Å] is *E*. An intra­molecular hy­droxy-O1—H⋯N2(imine) hydrogen bond is noted, Table 1 ▸. The dihedral angles between the central residue and the pyridyl and benzene rings are 23.16 (10) and 48.99 (9)°, respectively. As the six-membered rings are con-rotatory with respect to the chain, the dihedral angle between them of 71.79 (6)° indicates an approximately orthogonal relationship.

## Supra­molecular features   

**Figure 2:**
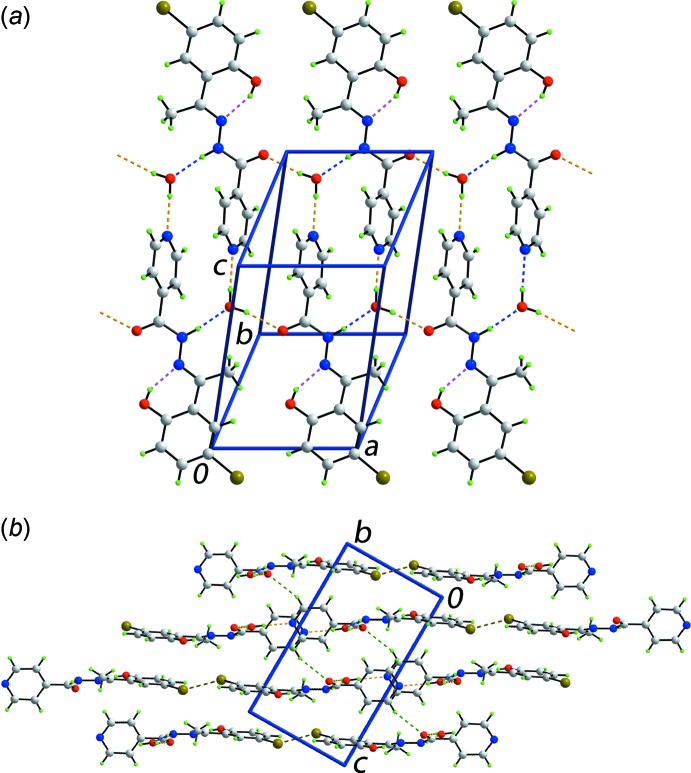
Mol­ecular packing in (I): (*a*) supra­molecular ribbons propagating along the *a* axis sustained by hydrazide-N—H⋯O(water) (shown as blue dashed lines), and water-O—H⋯O(carbon­yl) and water-O—H⋯N(pyrid­yl) hydrogen-bonds (orange dashed lines). Intra­molecular hy­droxy-O—H⋯N(imine) hydrogen-bonds are also indicated (pink dashed lines); (*b*) a view of the unit-cell contents in projection down the *a* axis. The Br⋯Br and C—H⋯O inter­actions are shown as olive-green and green dashed lines, respectively.

The most prominent feature of the supra­molecular association is the formation of supra­molecular ribbons, with a flat topology, parallel to (0
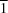
2), propagating along the *a-*axis direction and mediated by hydrogen-bonding inter­actions. In essence, the water mol­ecule provides links between three organic mol­ecules *via* hydrazide-N—H⋯O(water), water-O—H⋯O(carbon­yl) and water-O—H⋯N(pyrid­yl) hydrogen bonds, Table 1 ▸. This association leads to centrosymmetric, 18-membered {⋯HOH⋯NC_4_O}_2_ synthons as shown in Fig. 2 ▸ *a*. Lateral connections between ribbons are *via* halogen bonding of the type Br⋯Br. Here, the Br⋯Br^i^ separation is 3.5366 (3) Å [symmetry code: (i) −1 − *x*, 3 − *y*, 2 − *z*]. The C7—Br⋯Br^i^ angle is 156.56 (5)°, and, being disposed about a centre of inversion, the C7—Br⋯Br^i^—C7^i^ torsion angle is constrained by symmetry to 180°. The geometric characteristics indicate the Br⋯Br^i^ halogen bond is classified as a type I halogen bond (Desiraju & Parthasarathy, 1989 ▸). The connections between the layers are of the type pyridyl-C—H⋯O(carbon­yl), Table 1 ▸. These are reinforced by weak π–π inter­actions between inversion-related benzene rings: inter-centroid separation = 3.9315 (12) Å for symmetry operation −*x*, 2 − *y*, 2 − *z*.

## Hirshfeld surface analysis   

**Figure 3:**
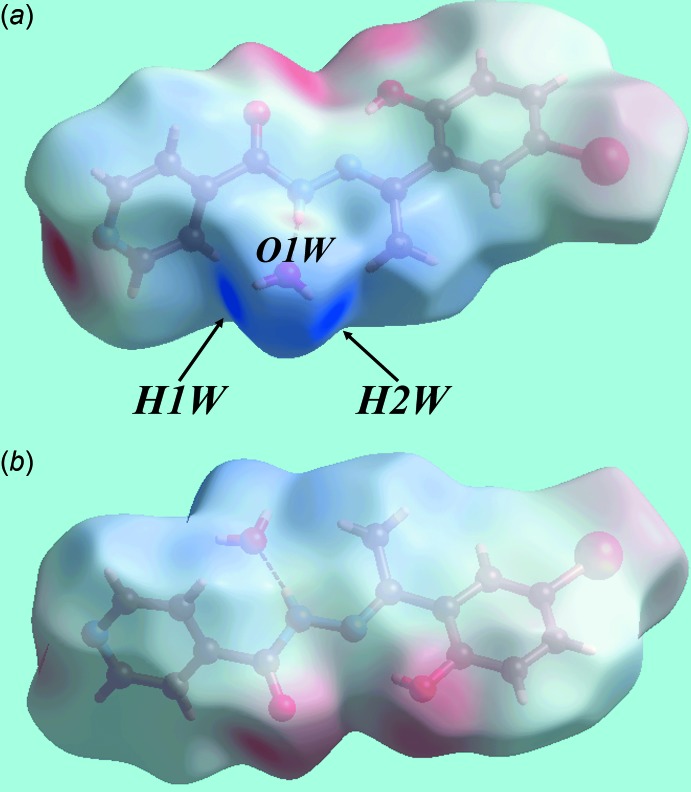
Views of the Hirshfeld surface for (I) mapped over the electrostatic potential over the range −0.122 to +0.156 au.

**Figure 4:**
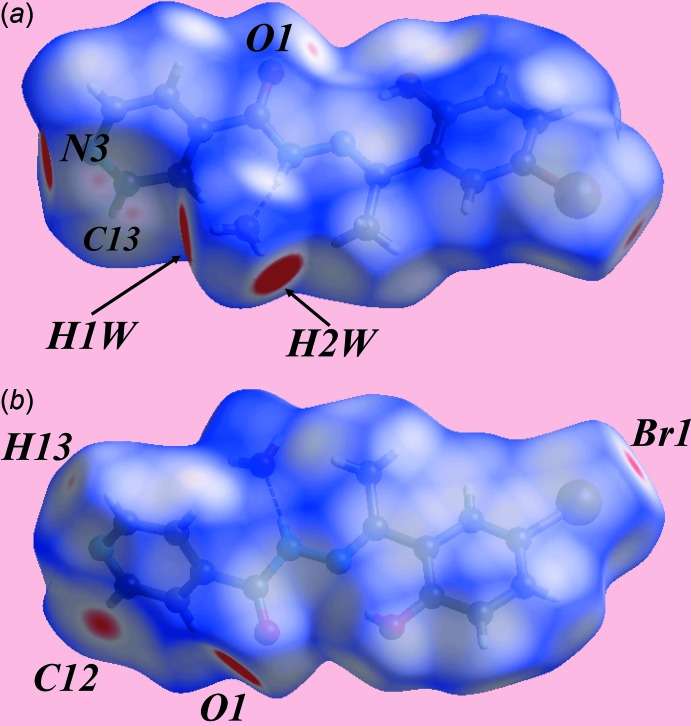
Views of the Hirshfeld surface for (I) mapped over *d*_norm_ over the range −0.150 to 1.528 au.

**Figure 5:**
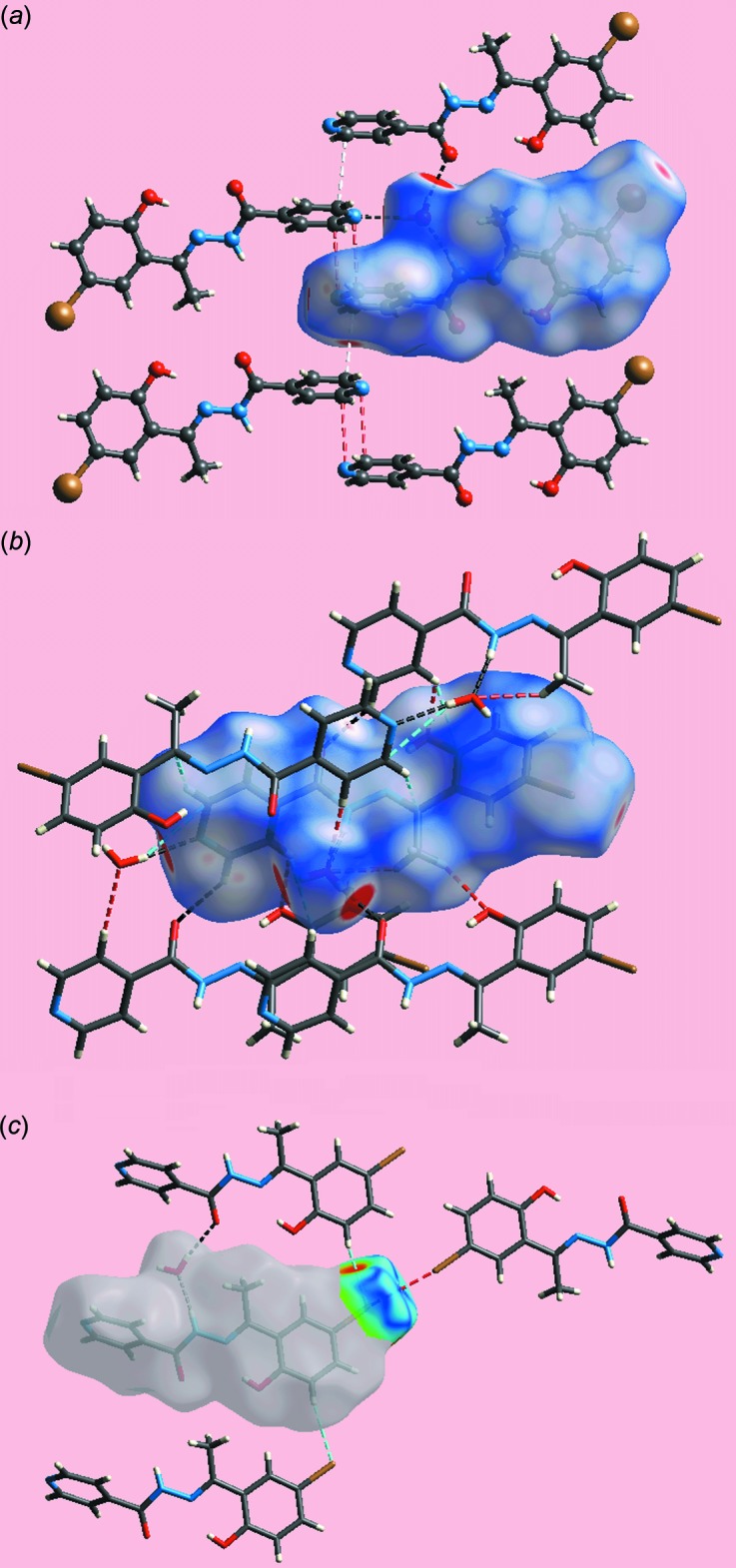
Views of the Hirshfeld surfaces, mapped over (*a*) and (*b*) *d*_norm_ and (*c*) shape-index, about a reference pair of mol­ecules comprising (I) highlighting short inter­atomic (*a*) C⋯H/H⋯C and O⋯H/H⋯O contacts through sky-blue and red dashed lines, respectively, (*b*) C⋯C and C⋯N/N⋯C contacts through white and red dashed lines, respectively, and (*c*) Br⋯Br and Br⋯H/H⋯Br contacts through red and sky-blue dashed lines, respectively.

The analysis of the Hirshfeld surface for (I) was performed as per a recent publication (Wardell *et al.*, 2016 ▸). Views of the Hirshfeld surface mapped over the calculated electrostatic potential are given in Fig. 3 ▸. It is important to note that despite its small size relative to the organic species, the presence of water in the crystal lattice exerts a great influence on the packing of (I) owing to the involvement of all of its atoms in conventional hydrogen bonds as well as short inter­atomic contacts (Table 2 ▸). This is also seen through the appearance in Fig. 3 ▸ *a* of a light-red spot (negative potential) within the surface near the water-O1*W* atoms as well as the blue regions (positive potential) about the water-H1*W* and H2*W* atoms, which correspond to the acceptor and donors of the hydrogen bonds, respectively. Similarly, the other donor and acceptor atoms participating in the more significant inter­molecular inter­actions are viewed as the blue and red regions, respectively, in Fig. 3 ▸. The donors and acceptors of water-O—H⋯O(carbon­yl) and water-O—H⋯N(pyrid­yl) hydrogen bonds on the Hirshfeld surfaces mapped over *d*_norm_ in Fig. 4 ▸ appear as bright-red spots near the respective atoms. The presence of red spots near the Br1 and pyridine-C12 atoms in Fig. 4 ▸ *b* also highlight the significant contribution of Br⋯Br and C⋯C contacts to the mol­ecular packing. The presence of faint-red spots near the pyridyl-N3, C13 and H13 atoms and the carbonyl-O1 atom indicate their contributions to short inter­atomic C⋯N/N⋯C contacts (Table 2 ▸) and comparatively weak inter­molecular C—H⋯O inter­actions, respectively. The immediate environments about a reference pair of mol­ecules comprising (I) within the *d*_norm_- (Fig. 5 ▸ *a* and *b*) and shape-index- (Fig. 5 ▸ *c*) mapped Hirshfeld surfaces highlighting the various short inter­atomic contacts influential on the mol­ecular packing are illustrated in Fig. 5 ▸. The C⋯H/H⋯C and O⋯H/H⋯O contacts, Fig. 5 ▸ *a*, C⋯C and C⋯N/N⋯C contacts, Fig. 5 ▸ *b*, and Br⋯Br and Br⋯H/H⋯Br contacts, Fig. 5 ▸ *c*, identify their roles in consolidating the packing in the crystal.

**Figure 6:**
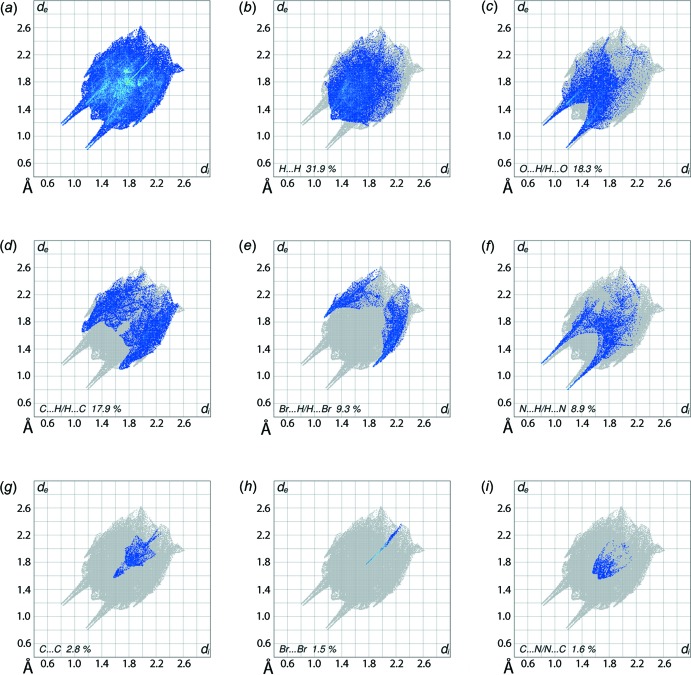
Fingerprint plots for (I): (*a*) overall and those delineated into (*b*) H⋯H, (*c*) O⋯H/H⋯O, (*d*) C⋯H/H⋯C, (*e*) Br⋯H/H⋯Br, (*f*) N⋯H/H⋯N, (*g*) C⋯C, (*h*) Br⋯Br and (*i*) N⋯C/C⋯N contacts.

The overall two-dimensional fingerprint plot, Fig. 6 ▸ *a*, and those delineated into H⋯H, O⋯H/H⋯O, C⋯H/H⋯C, Br⋯H/H⋯Br, N⋯H/H⋯N, C⋯C, Br⋯Br and C⋯N/N⋯C contacts (McKinnon *et al.*, 2007 ▸) are illustrated in Fig. 6 ▸ *b*–*i*, respectively; their relative contributions to the Hirshfeld surfaces are summarized in Table 3 ▸. The fingerprint plot delineated into H⋯H contacts, Fig. 6 ▸ *b*, shows that while these contacts have the greatest contribution to the Hirshfeld surface, *i.e*. 31.9%, due to the involvement of most of the hydrogen atoms of the mol­ecule in hydrogen bonds and short inter­atomic O⋯H/H⋯O and C⋯H/H⋯C contacts, there are relatively few hydrogen atoms available on the surface to form inter­atomic H⋯H contacts and, when in contact, are farther than the sum of their van der Waals radii. The pair of spikes with tips at *d*_e_ + *d*_i_ ∼ 2.0 Å in each of the fingerprint plots delineated into O⋯H/H⋯O contacts, Fig. 6 ▸ *c*, and N⋯H/H⋯N contacts, Fig. 6 ▸ *f*, arise as a result of O—H⋯O and O—H⋯N hydrogen bonds. As the Hirshfeld surfaces and two-dimensional fingerprint plots shown here are inclusive of the water mol­ecule, neither bright-red spots near the donor–acceptor atoms of hydrazine-N—H⋯O(water) hydrogen bonds are seen on the *d*_norm_-mapped Hirshfeld surface in Fig. 4 ▸ nor is there a pair of spikes on the corresponding fingerprint plot. Thus, the 18.3% contribution from O⋯H/H⋯O contacts to the surface results from the O—H⋯O hydrogen bonds and short inter­atomic contacts involving these atoms only (Table 2 ▸ and Fig. 5 ▸ *b*). The conformational relationship between each of the pyridyl and benzene rings to the central planar region make these residues available for forming C⋯H/H⋯C contacts. The significant contribution of 17.9% from C⋯H/H⋯C contacts results from the short inter­atomic contacts listed in Table 2 ▸, and appears as a symmetrical distribution of points showing characteristic wings in Fig. 6 ▸ *d* with the pair of peaks at *d*_e_ + *d*_i_ ∼ 2.8 Å; these short inter­atomic contacts are illustrated in Fig. 5 ▸ *a*. A forceps-like fingerprint plot corresponding to Br⋯H/H⋯Br contacts in Fig. 6 ▸ *e* with its tips at *d*_e_ + *d*_i_ ∼ 3.0 Å represents the influence of the halogen⋯hydrogen inter­actions in the mol­ecular packing. Along with Br⋯H/H⋯Br contacts, Table 2 ▸, the Br2 atom exerts an influence upon the mol­ecular packing *via* Br⋯Br contacts, as evident in Fig. 6 ▸ *h* as a very thin line beginning at *d*_e_ + *d*_i_ ∼ 3.5 Å. The contributions from other inter­atomic contacts involving the bromide atom have negligible effect on the crystal stability because their inter­atomic distances are much greater than sum of their respective van der Waals radii. The small but notable contributions from the C⋯C and C⋯N/N⋯C contacts to the Hirshfeld surface, Table 2 ▸, represent π–π stacking inter­actions. In Fig. 6 ▸ *g*, a spear-shaped distribution of points with the tip at *d*_e_ + *d*_i_ ∼ 3.2 Å and an adjacent arrow-like distribution of points at *d*_e_ = *d*_i_ ∼ 1.9 Å result, respectively, from inter­atomic C⋯C contacts and π–π stacking inter­actions involving the C3–C8 ring. The short inter­atomic C⋯N/N⋯C contacts involving the pyridyl-C13 and N3 atoms, Fig. 5 ▸ *b*, are reflected in a pair of small peaks at *d*_e_ + *d*_i_ ∼ 3.2 Å in Fig. 6 ▸ *i*. The small contributions from other inter­atomic contacts listed in Table 2 ▸ have a negligible effect on the overall packing of (I).

## Database survey   

**Figure 7:**
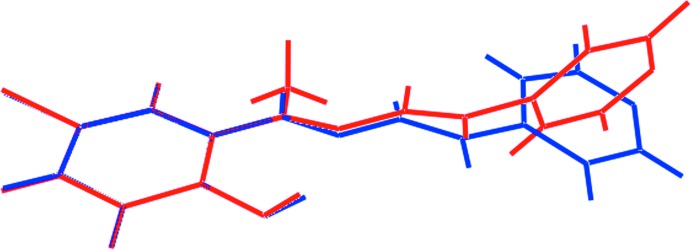
Overlay diagram of the organic mol­ecule in (I), red image, and (II), blue image. The mol­ecules are overlapped so the benzene rings are coincident.

**Figure 8:**
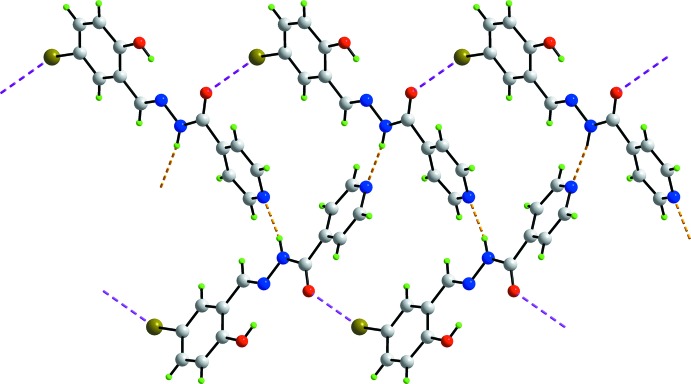
A view of the zigzag supra­molecular chain in (II) mediated by hydrazide-N—H⋯N(pyrid­yl) hydrogen bonds shown as blue dashed lines. The Br⋯O halogen bonds are indicated by purple dashed lines.

The most closely related structure to (I) in the crystallographic literature (Groom *et al.*, 2016 ▸) is one that lacks the imine-methyl substituent and is anhydrous, hereafter referred to as (II); a similar numbering scheme is adopted here. This structure has been reported twice (Yang, 2006 ▸; Sedaghat *et al.*, 2014 ▸) and data from the first determination are employed herein. Selected geometric parameters are collected in Table 4 ▸, from which it can be seen that there are no experimentally significant differences between the structures. However, there are conformational differences between the mol­ecules as highlighted in the overlay diagram shown in Fig. 7 ▸. While there is a close coincidence between the benzene rings and the first few atoms of the chain linking the rings, a twist occurs about the C1—C10 bond in (II), as seen in the N1—C1—C10–C14 torsion angle of 24.2 (5)°. The major consequence of this is seen in the dihedral angle between the rings of 11.23 (11)° *cf*. the near to orthogonal relationship in (I). This conformational difference likely relates to the distinct supra­molecular association in the crystals of (I) and (II). In (II), with no water mol­ecule to form hydrogen bonds, direct links between the organic mol­ecules are of the type hydrazide-N—H⋯N(pyrid­yl) and lead to zigzag supra­molecular chains, as illustrated in Fig. 8 ▸. Also evident from Fig. 8 ▸, is the close proximity of the bromide and oxygen atoms, which form type I Br⋯O halogen bonds, the separation between the atoms being 3.117 (3)°.

## Synthesis and crystallization   

All chemicals and solvents were used as purchased without purification, and all reactions were carried out under ambient conditions. The melting point was determined using an Electrothermal digital melting-point apparatus and was uncorrected. The IR spectrum for the compound was obtained on a Perkin Elmer Spectrum 400 FT Mid-IR/Far-IR spectrophotometer from 4000 to 400 cm^−1^. The ^1^H NMR spectrum was recorded at room temperature in CDCl_3_ solution on a Jeol ECA 400 MHz FT–NMR spectrometer.

1-(5-Bromo-2-hy­droxy­phen­yl)ethyl­idene]iso­nicotino­hydra­zide (1.0 mmol, 0.333 g) and tri­ethyl­amine (1.0 mmol, 0.14 ml) in methanol (25 ml) were added to di-*n*-butyl­tin dichloride (1.0 mmol, 0.303 g) in methanol (10 ml). The resulting mixture was stirred and refluxed for 3 h. A cloudy orange solution was obtained and the mixture was filtered. The filtrate was allowed to stand at room temperature and yellow crystals, suitable for X-ray crystallographic studies, were obtained after the slow evaporation. The yellow crystals were found to be a side-product from the reaction mixture. Yield: 0.112 g, 34%; M.p. 501 K. IR (cm^−1^): 3158(*br*), 1666(*s*), 1548(*s*), 1152 (*m*), 964(*s*) cm^−1. 1^H NMR (in CDCl_3_): 11.20 (*s*, 1H, NH), 8.73-8.82, 7.92-8.20, 6.80-6.99 (*m*, 7H, aromatic-H), 4.82 (*br*, 2H, H_2_O), 4.10 (*br*, 1H, OH), 3.13 (*s*, 3H, –CH_3_).

## Refinement details   

Crystal data, data collection and structure refinement details are summarized in Table 5 ▸. Carbon-bound H atoms were placed in calculated positions (C—H = 0.99–1.00 Å) and were included in the refinement in the riding-model approximation, with *U*_iso_(H) set to 1.2*U*_eq_(C).

## Supplementary Material

Crystal structure: contains datablock(s) I, global. DOI: 10.1107/S2056989017004790/hb7669sup1.cif

Structure factors: contains datablock(s) I. DOI: 10.1107/S2056989017004790/hb7669Isup2.hkl

Supporting information file. DOI: 10.1107/S2056989017004790/hb7669Isup3.cml

CCDC reference: 1540550

Additional supporting information:  crystallographic information; 3D view; checkCIF report

## Figures and Tables

**Table 1:** Hydrogen-bond geometry (Å, °)

*D*—H⋯*A*	*D*—H	H⋯*A*	*D*⋯*A*	*D*—H⋯*A*
O2—H2*O*⋯N2	0.75 (3)	1.87 (3)	2.552 (2)	152 (3)
N1—H1*N*⋯O1*W*	0.88 (2)	1.91 (2)	2.779 (2)	170 (2)
O1*W*—H1*W*⋯N3^i^	0.84 (2)	1.98 (2)	2.822 (2)	176 (3)
O1*W*—H2*W*⋯O1^ii^	0.84 (2)	2.00 (2)	2.828 (2)	171 (2)
C13—H13⋯O1^iii^	0.95	2.54	3.387 (3)	148

Symmetry codes: (i) 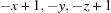; (ii) 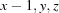; (iii) 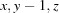.

**Table 2:** Summary of short inter­atomic contacts (Å) in (I)

Contact	distance	symmetry operation
Br1⋯Br1	3.5366 (3)	−1 − *x*, 3 − *y*, 2 − *z*
C12⋯C12	3.161 (3)	2 − *x*, −*y*, 1 − *z*
N3⋯C13	3.207 (3)	1 − *x*, −*y*, 1 − *z*
C9⋯O1*W*	3.168 (2)	*x*, *y*, *z*
Br1⋯H5	3.02	−1 + *x*, *y*, *z*
O1*W*⋯H9*C*	2.62	*x*, *y*, *z*
O1*W*⋯H11	2.65	1 − *x*, 1 − *y*, 1 − *z*
O2⋯H9*B*	2.63	1 + *x*, *y*, *z*
O2⋯H14	2.66	*x*, 1 + *y*, *z*
C2⋯H12	2.85	1 − *x*, 1 − *y*, 1 − *z*
C4⋯H14	2.77	*x*, 1 + *y*, *z*
C12⋯H1*W*	2.84 (2)	1 − *x*, −*y*, 1 − *z*

**Table 3:** Percentage contribution to inter­atomic contacts from the Hirshfeld surface for (I)

Contact	percentage contribution
H⋯H	31.9
O⋯H/H⋯O	18.3
C⋯H/H⋯C	17.9
Br⋯H/H⋯Br	9.3
N⋯H/H⋯N	8.9
Br⋯C/C⋯Br	3.1
C⋯C	2.8
Br⋯N/N⋯Br	2.3
C⋯N / N⋯C	1.6
Br⋯Br	1.5
Br⋯O/O⋯Br	1.5
C⋯O/O⋯C	0.8
N⋯N	0.1

**Table 4:** Selected geometric parameters (Å, °) for (I) and (II)

Parameter	(I)	(II)^*a*^
N1—N2	1.375 (2)	1.369 (4)
C1—O1	1.225 (2)	1.204 (4)
C1—N1	1.362 (2)	1.353 (4)
C2—N2	1.292 (2)	1.270 (4)
C4—O2	1.355 (2)	1.352 (3)
Br1–C7	1.9084 (17)	1.895 (3)

Notes: (*a*) Yang (2006 ▸).

**Table 5:** Experimental details

Crystal data	
Chemical formula	C_14_H_12_BrN_3_O_2_·H_2_O
*M*_r_	352.19
Crystal system, space group	Triclinic, *P* 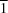
Temperature (K)	100
*a*, *b*, *c* (Å)	7.1123 (2), 7.7841 (2), 13.3011 (5)
α, β, γ (°)	87.604 (3), 84.299 (3), 72.447 (3)
*V* (Å^3^)	698.57 (4)
*Z*	2
Radiation type	Cu *K*α
μ (mm^−1^)	4.15
Crystal size (mm)	0.29 × 0.18 × 0.04

Data collection	
Diffractometer	Agilent SuperNova, Dual, Cu at zero, AtlasS2
Absorption correction	Multi-scan (*CrysAlis PRO*; Rigaku Oxford Diffraction, 2015 ▸)
*T*_min_, *T*_max_	0.652, 1.000
No. of measured, independent and observed [*I* > 2σ(*I*)] reflections	12875, 2774, 2679
*R*_int_	0.035
(sin θ/λ)_max_ (Å^−1^)	0.625

Refinement	
*R*[*F* ^2^ > 2σ(*F* ^2^)], *wR*(*F* ^2^), *S*	0.027, 0.073, 1.06
No. of reflections	2774
No. of parameters	203
No. of restraints	3
H-atom treatment	H-atom parameters not refined
Δρ_max_, Δρ_min_ (e Å^−3^)	0.73, −0.45

Computer programs: *CrysAlis PRO* (Rigaku Oxford Diffraction, 2015 ▸), *SHELXS* (Sheldrick, 2008 ▸), *SHELXL2014/7* (Sheldrick, 2015 ▸), *ORTEP-3 for Windows* (Farrugia, 2012 ▸), *QMol* (Gans & Shalloway, 2001 ▸), *DIAMOND* (Brandenburg, 2006 ▸) and *publCIF* (Westrip, 2010 ▸).

## supplementary crystallographic information

### Crystal data

**Table:**

C_14_H_12_BrN_3_O_2_·H_2_O	*Z* = 2
*M**_r_* = 352.19	*F*(000) = 356
Triclinic, *P*1	*D*_x_ = 1.674 Mg m^−^^3^
*a* = 7.1123 (2) Å	Cu *K*α radiation, λ = 1.54184 Å
*b* = 7.7841 (2) Å	Cell parameters from 8299 reflections
*c* = 13.3011 (5) Å	θ = 3.3–73.7°
α = 87.604 (3)°	µ = 4.15 mm^−^^1^
β = 84.299 (3)°	*T* = 100 K
γ = 72.447 (3)°	Plate, yellow
*V* = 698.57 (4) Å^3^	0.29 × 0.18 × 0.04 mm

### Data collection

**Table:**

Agilent SuperNova, Dual, Cu at zero, AtlasS2 diffractometer	2774 independent reflections
Radiation source: micro-focus sealed X-ray tube, SuperNova (Cu) X-ray Source	2679 reflections with *I* > 2σ(*I*)
Mirror monochromator	*R*_int_ = 0.035
ω scans	θ_max_ = 74.5°, θ_min_ = 3.3°
Absorption correction: multi-scan (CrysAlisPro; Rigaku Oxford Diffraction, 2015)	*h* = −8→8
*T*_min_ = 0.652, *T*_max_ = 1.000	*k* = −9→9
12875 measured reflections	*l* = −15→16

### Refinement

**Table:**

Refinement on *F*^2^	3 restraints
Least-squares matrix: full	Hydrogen site location: inferred from neighbouring sites
*R*[*F*^2^ > 2σ(*F*^2^)] = 0.027	H-atom parameters not refined
*wR*(*F*^2^) = 0.073	*w* = 1/[σ^2^(*F*_o_^2^) + (0.0482*P*)^2^ + 0.327*P*] where *P* = (*F*_o_^2^ + 2*F*_c_^2^)/3
*S* = 1.06	(Δ/σ)_max_ = 0.002
2774 reflections	Δρ_max_ = 0.73 e Å^−^^3^
203 parameters	Δρ_min_ = −0.45 e Å^−^^3^

### Special details

**Table:**

Geometry. All esds (except the esd in the dihedral angle between two l.s. planes) are estimated using the full covariance matrix. The cell esds are taken into account individually in the estimation of esds in distances, angles and torsion angles; correlations between esds in cell parameters are only used when they are defined by crystal symmetry. An approximate (isotropic) treatment of cell esds is used for estimating esds involving l.s. planes.

### Fractional atomic coordinates and isotropic or equivalent isotropic displacement parameters (Å^2^)

**Table:**

	*x*	*y*	*z*	*U*_iso_*/*U*_eq_	
Br1	−0.28431 (2)	1.37135 (2)	0.92836 (2)	0.01956 (9)	
O1	0.7358 (2)	0.50978 (18)	0.66291 (11)	0.0213 (3)	
O2	0.53522 (19)	0.85281 (19)	0.85029 (11)	0.0193 (3)	
H2O	0.528 (4)	0.777 (4)	0.819 (2)	0.029*	
N1	0.4214 (2)	0.4971 (2)	0.70654 (13)	0.0169 (3)	
N2	0.3809 (2)	0.6565 (2)	0.75717 (12)	0.0169 (3)	
H1N	0.330 (3)	0.457 (3)	0.6853 (18)	0.020*	
N3	0.7466 (2)	−0.0677 (2)	0.50649 (13)	0.0205 (3)	
C1	0.6085 (3)	0.4338 (2)	0.66074 (15)	0.0168 (4)	
C2	0.2022 (3)	0.7409 (2)	0.79238 (14)	0.0157 (4)	
C3	0.1803 (3)	0.9134 (2)	0.84109 (14)	0.0157 (4)	
C4	0.3461 (3)	0.9597 (2)	0.86858 (14)	0.0167 (4)	
C5	0.3202 (3)	1.1216 (3)	0.91635 (15)	0.0190 (4)	
H5	0.4320	1.1492	0.9367	0.023*	
C6	0.1337 (3)	1.2432 (2)	0.93470 (15)	0.0191 (4)	
H6	0.1169	1.3542	0.9668	0.023*	
C7	−0.0282 (3)	1.2004 (2)	0.90563 (14)	0.0163 (4)	
C8	−0.0088 (3)	1.0386 (2)	0.86125 (14)	0.0172 (4)	
H8	−0.1230	1.0111	0.8442	0.021*	
C9	0.0260 (3)	0.6761 (2)	0.78392 (16)	0.0192 (4)	
H9A	−0.0276	0.7156	0.7189	0.029*	
H9B	−0.0757	0.7262	0.8390	0.029*	
H9C	0.0662	0.5442	0.7884	0.029*	
C10	0.6498 (3)	0.2595 (2)	0.60670 (15)	0.0168 (4)	
C11	0.7443 (3)	0.2420 (3)	0.50953 (15)	0.0191 (4)	
H11	0.7793	0.3399	0.4763	0.023*	
C12	0.7863 (3)	0.0775 (3)	0.46231 (16)	0.0211 (4)	
H12	0.8465	0.0668	0.3949	0.025*	
C13	0.6583 (3)	−0.0494 (3)	0.60073 (16)	0.0202 (4)	
H13	0.6308	−0.1512	0.6334	0.024*	
C14	0.6051 (3)	0.1114 (2)	0.65315 (15)	0.0191 (4)	
H14	0.5396	0.1200	0.7194	0.023*	
O1W	0.1501 (2)	0.3754 (2)	0.61615 (13)	0.0287 (3)	
H1W	0.184 (5)	0.286 (3)	0.5776 (19)	0.043*	
H2W	0.0259 (15)	0.409 (4)	0.624 (2)	0.043*	

### Atomic displacement parameters (Å^2^)

**Table:**

	*U*^11^	*U*^22^	*U*^33^	*U*^12^	*U*^13^	*U*^23^
Br1	0.01371 (12)	0.01378 (12)	0.02867 (14)	−0.00048 (8)	0.00004 (8)	−0.00385 (8)
O1	0.0149 (6)	0.0178 (6)	0.0320 (8)	−0.0063 (5)	0.0013 (5)	−0.0063 (5)
O2	0.0114 (6)	0.0165 (6)	0.0298 (8)	−0.0029 (5)	−0.0014 (5)	−0.0068 (5)
N1	0.0128 (7)	0.0141 (7)	0.0244 (8)	−0.0041 (6)	−0.0011 (6)	−0.0057 (6)
N2	0.0150 (7)	0.0130 (7)	0.0220 (8)	−0.0026 (6)	−0.0016 (6)	−0.0042 (6)
N3	0.0143 (7)	0.0171 (7)	0.0296 (9)	−0.0025 (6)	−0.0046 (6)	−0.0048 (6)
C1	0.0138 (8)	0.0153 (8)	0.0208 (9)	−0.0032 (7)	−0.0033 (7)	0.0001 (7)
C2	0.0135 (8)	0.0147 (8)	0.0184 (9)	−0.0031 (7)	−0.0024 (7)	−0.0001 (7)
C3	0.0137 (8)	0.0142 (8)	0.0189 (9)	−0.0042 (7)	−0.0003 (7)	−0.0005 (7)
C4	0.0129 (8)	0.0164 (8)	0.0203 (9)	−0.0039 (7)	−0.0001 (7)	0.0003 (7)
C5	0.0153 (9)	0.0182 (9)	0.0249 (10)	−0.0069 (7)	−0.0012 (7)	−0.0027 (7)
C6	0.0212 (9)	0.0141 (8)	0.0224 (10)	−0.0060 (7)	−0.0007 (7)	−0.0029 (7)
C7	0.0126 (8)	0.0133 (8)	0.0205 (9)	−0.0009 (6)	0.0020 (7)	−0.0026 (7)
C8	0.0139 (8)	0.0160 (8)	0.0220 (9)	−0.0054 (7)	−0.0005 (7)	−0.0009 (7)
C9	0.0139 (8)	0.0152 (8)	0.0288 (10)	−0.0049 (7)	0.0010 (7)	−0.0064 (7)
C10	0.0118 (8)	0.0144 (8)	0.0235 (9)	−0.0021 (6)	−0.0029 (7)	−0.0031 (7)
C11	0.0154 (8)	0.0175 (9)	0.0240 (10)	−0.0042 (7)	−0.0015 (7)	−0.0003 (7)
C12	0.0167 (9)	0.0211 (9)	0.0238 (10)	−0.0030 (7)	−0.0010 (7)	−0.0044 (7)
C13	0.0157 (9)	0.0161 (8)	0.0287 (10)	−0.0042 (7)	−0.0029 (7)	−0.0006 (7)
C14	0.0158 (9)	0.0171 (9)	0.0241 (10)	−0.0047 (7)	−0.0009 (7)	−0.0013 (7)
O1W	0.0143 (7)	0.0285 (8)	0.0436 (9)	−0.0054 (6)	0.0008 (6)	−0.0199 (7)

### Geometric parameters (Å, º)

**Table:**

Br1—C7	1.9084 (17)	C6—C7	1.384 (3)
O1—C1	1.225 (2)	C6—H6	0.9500
O2—C4	1.355 (2)	C7—C8	1.377 (3)
O2—H2O	0.75 (3)	C8—H8	0.9500
N1—C1	1.362 (2)	C9—H9A	0.9800
N1—N2	1.375 (2)	C9—H9B	0.9800
N1—H1N	0.876 (10)	C9—H9C	0.9800
N2—C2	1.292 (2)	C10—C11	1.388 (3)
N3—C13	1.338 (3)	C10—C14	1.391 (3)
N3—C12	1.344 (3)	C11—C12	1.387 (3)
C1—C10	1.497 (3)	C11—H11	0.9500
C2—C3	1.475 (3)	C12—H12	0.9500
C2—C9	1.500 (3)	C13—C14	1.390 (3)
C3—C8	1.410 (3)	C13—H13	0.9500
C3—C4	1.417 (3)	C14—H14	0.9500
C4—C5	1.389 (3)	O1W—H1W	0.844 (10)
C5—C6	1.383 (3)	O1W—H2W	0.839 (10)
C5—H5	0.9500		
			
C4—O2—H2O	105 (2)	C6—C7—Br1	118.68 (14)
C1—N1—N2	115.40 (15)	C7—C8—C3	120.15 (18)
C1—N1—H1N	117.6 (16)	C7—C8—H8	119.9
N2—N1—H1N	123.5 (17)	C3—C8—H8	119.9
C2—N2—N1	120.71 (16)	C2—C9—H9A	109.5
C13—N3—C12	117.39 (17)	C2—C9—H9B	109.5
O1—C1—N1	123.98 (18)	H9A—C9—H9B	109.5
O1—C1—C10	121.51 (17)	C2—C9—H9C	109.5
N1—C1—C10	114.51 (16)	H9A—C9—H9C	109.5
N2—C2—C3	114.57 (17)	H9B—C9—H9C	109.5
N2—C2—C9	124.46 (17)	C11—C10—C14	118.92 (18)
C3—C2—C9	120.96 (16)	C11—C10—C1	119.27 (16)
C8—C3—C4	117.92 (17)	C14—C10—C1	121.72 (17)
C8—C3—C2	120.36 (17)	C12—C11—C10	118.14 (17)
C4—C3—C2	121.72 (16)	C12—C11—H11	120.9
O2—C4—C5	116.47 (17)	C10—C11—H11	120.9
O2—C4—C3	123.21 (17)	N3—C12—C11	123.72 (18)
C5—C4—C3	120.32 (17)	N3—C12—H12	118.1
C6—C5—C4	120.84 (18)	C11—C12—H12	118.1
C6—C5—H5	119.6	N3—C13—C14	123.11 (17)
C4—C5—H5	119.6	N3—C13—H13	118.4
C5—C6—C7	118.98 (18)	C14—C13—H13	118.4
C5—C6—H6	120.5	C13—C14—C10	118.67 (18)
C7—C6—H6	120.5	C13—C14—H14	120.7
C8—C7—C6	121.74 (17)	C10—C14—H14	120.7
C8—C7—Br1	119.58 (14)	H1W—O1W—H2W	106 (3)
			
C1—N1—N2—C2	171.14 (18)	C5—C6—C7—Br1	−178.54 (14)
N2—N1—C1—O1	0.0 (3)	C6—C7—C8—C3	−2.2 (3)
N2—N1—C1—C10	179.59 (15)	Br1—C7—C8—C3	177.99 (14)
N1—N2—C2—C3	−177.59 (15)	C4—C3—C8—C7	0.5 (3)
N1—N2—C2—C9	1.0 (3)	C2—C3—C8—C7	−179.21 (17)
N2—C2—C3—C8	165.02 (17)	O1—C1—C10—C11	−47.1 (3)
C9—C2—C3—C8	−13.6 (3)	N1—C1—C10—C11	133.33 (18)
N2—C2—C3—C4	−14.6 (3)	O1—C1—C10—C14	129.6 (2)
C9—C2—C3—C4	166.75 (17)	N1—C1—C10—C14	−50.0 (3)
C8—C3—C4—O2	−177.99 (17)	C14—C10—C11—C12	1.3 (3)
C2—C3—C4—O2	1.7 (3)	C1—C10—C11—C12	178.06 (17)
C8—C3—C4—C5	1.7 (3)	C13—N3—C12—C11	1.2 (3)
C2—C3—C4—C5	−178.61 (17)	C10—C11—C12—N3	−2.3 (3)
O2—C4—C5—C6	177.42 (17)	C12—N3—C13—C14	0.8 (3)
C3—C4—C5—C6	−2.3 (3)	N3—C13—C14—C10	−1.7 (3)
C4—C5—C6—C7	0.6 (3)	C11—C10—C14—C13	0.5 (3)
C5—C6—C7—C8	1.6 (3)	C1—C10—C14—C13	−176.15 (17)

### Hydrogen-bond geometry (Å, º)

**Table:**

*D*—H···*A*	*D*—H	H···*A*	*D*···*A*	*D*—H···*A*
O2—H2*O*···N2	0.75 (3)	1.87 (3)	2.552 (2)	152 (3)
N1—H1*N*···O1*W*	0.88 (2)	1.91 (2)	2.779 (2)	170 (2)
O1*W*—H1*W*···N3^i^	0.84 (2)	1.98 (2)	2.822 (2)	176 (3)
O1*W*—H2*W*···O1^ii^	0.84 (2)	2.00 (2)	2.828 (2)	171 (2)
C13—H13···O1^iii^	0.95	2.54	3.387 (3)	148

Symmetry codes: (i) −*x*+1, −*y*, −*z*+1; (ii) *x*−1, *y*, *z*; (iii) *x*, *y*−1, *z*.

## References

[bb1] Brandenburg, K. (2006). *DIAMOND*. Crystal Impact GbR, Bonn, Germany.

[bb2] Davies, A. G., Gielen, M., Pannell, K. H. & Tiekink, E. R. T. (2008). *Tin Chemistry, Fundamentals, Frontiers, and Applications*. Chichester: John Wiley & Sons Ltd.

[bb3] Desiraju, G. R. & Parthasarathy, R. (1989). *J. Am. Chem. Soc.* **111**, 8725–8726.

[bb4] Farrugia, L. J. (2012). *J. Appl. Cryst.* **45**, 849–854.

[bb5] Gans, J. & Shalloway, D. (2001). *J. Mol. Graphics Modell.* **19**, 557–559. 10.1016/s1093-3263(01)00090-0 11552684

[bb6] Groom, C. R., Bruno, I. J., Lightfoot, M. P. & Ward, S. C. (2016). *Acta Cryst.* B**72**, 171–179. 10.1107/S2052520616003954 PMC4822653 27048719

[bb7] Lee, S. M., Mohd Ali, H., Sim, K. S., Abdul Malek, S. N. & Lo, K. M. (2013). *Inorg. Chim. Acta*, **406**, 272–278.

[bb8] Lee, S. M., Mohd Ali, H., Sim, K. S., Abdul Malek, S. N. & Lo, K. M. (2012). *Appl. Organomet. Chem.* **26**, 310–319.

[bb9] Lee, S. M., Sim, K. S. & Lo, K. M. (2015). *Inorg. Chim. Acta*, **429**, 195–208.

[bb10] McKinnon, J. J., Jayatilaka, D. & Spackman, M. A. (2007). *Chem. Commun.* pp. 3814–3816. 10.1039/b704980c 18217656

[bb11] Nie, A. & Huang, Z. (2006). *J. Comb. Chem.* **8**, 655–658. 10.1021/cc060085l 16961402

[bb12] Rigaku Oxford Diffraction (2015). *CrysAlis PRO*. Agilent Technologies Inc., Santa Clara, CA, USA.

[bb13] Sedaghat, T., Yousefi, M., Bruno, G., Rudbari, H. A., Motamedi, H. & Nobakht, V. (2014). *Polyhedron*, **79**, 88–96.

[bb14] Sheldrick, G. M. (2008). *Acta Cryst.* A**64**, 112–122. 10.1107/S0108767307043930 18156677

[bb15] Sheldrick, G. M. (2015). *Acta Cryst.* C**71**, 3–8.

[bb16] Wardell, J. L., Jotani, M. M. & Tiekink, E. R. T. (2016). *Acta Cryst.* E**72**, 1618–1627. 10.1107/S2056989016016492 PMC5095847 27840722

[bb17] Wei, Z., Wang, J., Jiang, X., Li, Y., Chen, G. & Xie, Q. (2015). *Chin. J. Appl. Chem.* **32**, 1014–1020.

[bb18] Westrip, S. P. (2010). *J. Appl. Cryst.* **43**, 920–925.

[bb19] Yang, D.-S. (2006). *Acta Cryst.* E**62**, o3792–o3793.

